# Evaluation of Different Concentrations of Antimicrobial Quaternary Polymers on the Behavior of Gelatin- and Starch-Based Films

**DOI:** 10.3390/polym16223168

**Published:** 2024-11-14

**Authors:** Celeste Cottet, M. Fernández-García, M. A. Peltzer

**Affiliations:** 1 Laboratory of Obtention, Modification, Characterization and Evaluation of Materials (LOMCEM), Department of Science and Technology, University of Quilmes, Buenos Aires B1876BXD, Argentina; mercedes.peltzer@unq.edu.ar; 2 National Scientific and Technical Research Council (CONICET), Buenos Aires C1033AAJ, Argentina; 3 Institute of Polymer Science and Technology, Superior Council of Scientific Investigations (ICTP-CSIC), 28006 Madrid, Spain; martafg@ictp.csic.es; 4 Interdisciplinary Platform for Sustainable Plastics towards a Circular Economy, SUSPLAST, CSIC, 28006 Madrid, Spain

**Keywords:** poly(itaconic acid), quaternized polymers, gelatin, starch, active packaging

## Abstract

Nowadays, incorporating quaternary ammonium groups into polymers is one of the most promising strategies for preparing antimicrobial biomaterials for general applications. The main objective of this work was to evaluate the effect of different concentrations of antimicrobial quaternary polymers in gelatin- and starch-based films for the development of active materials intended for applications in food packaging and medical fields. Two antimicrobial biobased polymers, called MeFPIAx (MeFPIA1 and MeFPIA2), were previously synthesized through the radical polymerization of itaconic acid (IA), followed by their subsequent functionalization and modification. Both polymers were incorporated into a new blend of gelatin and starch (15% *w*/*w*, 4:1 mass ratio), using glycerol (30% *w*/*w*) as a plasticizer. Films were prepared using the casting technique from aqueous dispersions of the polymers and their structure was characterized by Fourier Transform Infrared Spectroscopy with Attenuated Total Reflectance (FTIR-ATR). The findings of this study showed the addition of MeFPIAx had a significant effect (*p* < 0.05), resulting in films with higher tensile strength (TS) and a higher Young’s modulus (YM), with values close to 20 MPa and exceeding 250 MPa, respectively. On the other hand, elongation at break (EB) values lower than 80% were obtained. Additionally, the swelling was reduced from ~400% to 100% and a reduction in water vapor permeability (P_w_) was observed, thanks to the increased interaction between the polymeric chains. Differential scanning calorimetry (DSC) scans showed that the addition of MeFPIAx increased the glass transition temperatures (T_g_) from 29 °C to 65 °C. Furthermore, thermogravimetry analysis (TGA) indicated an increase in the initial degradation temperatures, suggesting that the films were more thermally resistant. Finally, the films exhibited slight antioxidant activity but significant antimicrobial activity, achieving bacterial reduction values greater than 70% with the incorporation of MeFPIAx polymers against Gram-positive *Staphylococcus aureus*.

## 1. Introduction

Microorganisms are an integral part of our lives; however, some can unfavorably influence the health and well-being of plants, animals, and humans, causing serious problems. Nowadays, the main concerns of modern society are infectious diseases and health-related issues [1]. Infectious diseases, primarily caused by bacteria, account for approximately a quarter of all deaths worldwide and are responsible for the majority of infections in surgical procedures [2]. Furthermore, damage caused by food spoilage and contamination due to microbial activity deteriorates sensory attributes and affects both the food industry (economic losses, reputational damage, etc.) and consumers (product rejection) [3]. While antibiotics revolutionized healthcare in the last century by addressing major health issues, their overuse has led microorganisms to mutate, resulting in antibiotic resistance and, consequently, the uncontrolled spread of pathogens [4,5]. Therefore, it is evident that there is a constant need to develop new antimicrobial agents. In this context, antimicrobial polymers offer great versatility in preventing microbial contaminations or infections, particularly those containing quaternary ammonium (QA) groups [6]. It is known that QA polymers destabilize the cytoplasmic membrane of bacteria cells, which eventually leads to cell death [2,5,7,8]. The cell wall structure of Gram-positive and Gram-negative bacteria differs. The difference lies in the fact that Gram-negative bacteria have an outer membrane in the cell wall, composed of lipopolysaccharides. Due to its unique properties, this structure generally limits the penetration of various antimicrobial agents and amphiphilic compounds into the cell, so their activity depends on both their structure and the type of microorganism they interact with [9]. Lately, the application of QA polymers has gained the attention of researchers because of the effectiveness of these ionic compounds in forming intermolecular interactions with natural polymers for developing antimicrobial biomaterials [10]. Many of them are not only antibacterial but also possess antifungal, antiviral, and anti-matrix metalloproteinase or matrixin capabilities [11].

The present study is based on a previous research line focused on the synthesis of quaternary polymers with different degrees of functionalization of poly(itaconic) acid (PIA) that have been incorporated into natural polymer matrices, for the development of environmentally friendly materials [8]. Gelatin and starch are considered suitable alternatives to replace those materials of petrochemical origin, which is another one of today’s issues [12,13]. The novelty of the published work was to obtain a cationic system where the migration of the active agent to the surrounding media does not present a problem [8]. Consequently, two categories of antimicrobial materials can be distinguished. One is the materials where the active agents, such as Ag, Cu ions, essential oils, or antibiotics, among others, are released from the surface [14,15] to a specific target. In this case, the content of the antimicrobial agent is limited, and it should be considered that the release rate is not easily controllable. Hence, once the concentration of the active compound decreases, the material is not able to exhibit antimicrobial activity, so a long-term antibacterial effect cannot be achieved [9]. The second group is the contact antimicrobial films [16,17] that, compared to release materials, the antimicrobial agents is covalently bound to the polymer structure. Therefore, the direct-contact elimination approach is advantageous because (1) it enhances and extends antimicrobial activity, and (2) unlike antibiotics, its mode of action disrupts bacterial cell walls and membranes, making the development of antibiotic resistance improbable [7,9,18].

In our previous work, we described the synthesis of two cationic polymers, MeFPIA1 and MeFPIA2, and their incorporation into a natural matrix at a concentration of 10% wt [8]. In that work, the resulting films presented good results in terms of antimicrobial and antioxidant activities. However, the remaining question was what would happen if higher concentrations of the polymer and a lower concentration of the plasticizer were incorporated into the matrix: would the activities be improved? Therefore, this work aimed to study the addition of different concentrations of MeFPIAx into gelatin- and starch-based films to make an adequate adjustment to their formulation and determine the precise concentration of the active agent and plasticizer to be incorporated into the formulation.

## 2. Materials and Methods

### 2.1. Materials

Gelatin (Scharlau^®^, Barcelona, Spain), starch (Nestlé Health Science^®^, Barcelona, Spain), glycerol (≥99%, Sigma Aldrich^®^, St. Louis, MO, USA), sodium hydroxide (NaOH, ≥98%, Sigma-Aldrich^®^), potassium carbonate (K_2_CO_3_, Sigma-Aldrich^®^), magnesium nitrate (Mg(NO_3_)_2_, 98%, Sigma-Aldrich^®^), barium chloride (BaCl_2_, ≥98%, Sigma-Aldrich^®^), phosphate-buffered saline (PBS, pH 7.4, Sigma-Aldrich^®^), 2,2′-azino-bis(3-ethylbenzothiazoline-6-sulfonic acid) diammonium salt (ABTS, Sigma^®^, Tokyo, Japan), potassium persulfate (K_2_S_2_O_8_, Sigma^®^), 2,2-diphenyl-1-picrylhydrazyl (DPPH, Sigma^®^), 96% *v/v* ethanol (Porta^®^, Kyoto, Japan), peptone (Britania^®^, Kolkata, India), bacteriological agar (Britania^®^), nutrient broth (Britania^®^).

### 2.2. Preparation of Gelatin- and Starch-Based Films with Quaternary Polymers, MeFPIAx

**Figure 1:**
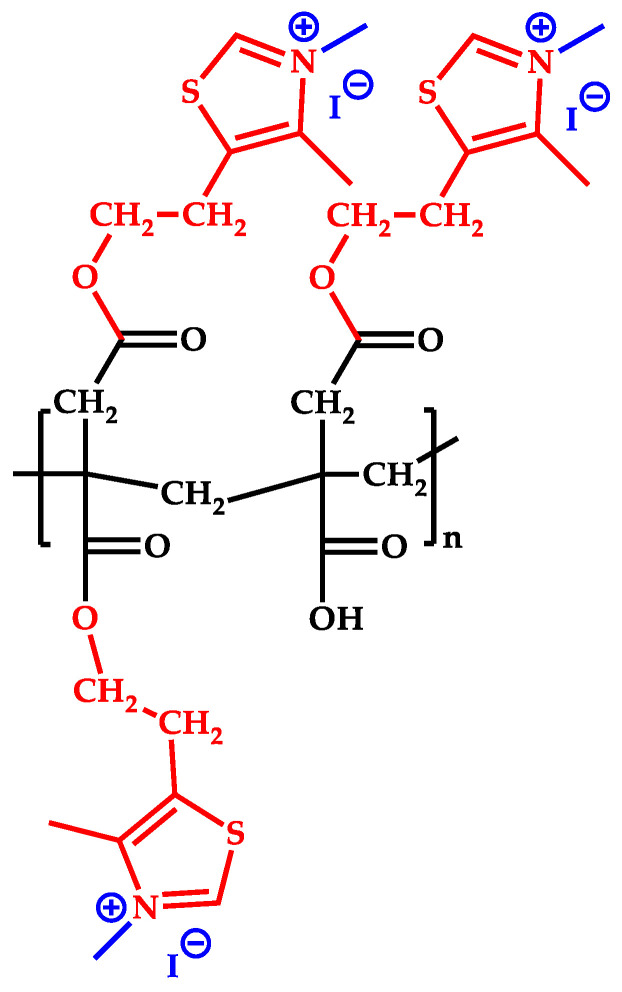
The structure of the synthesized polymers MeFPIAx.

Briefly, poly(itaconic acid) (PIA) was obtained via radical polymerization of itaconic acid in water with ammonium persulfate as the initiator at 60 °C for 48 h. The functionalization of PIA (FPIA) by esterification with the 1,3-thiazole heterocycle groups was carried out with an excess of this compound (1:4 molar ratio) using H_2_SO_4_ as a catalyst at 90 °C and 120 °C for 24 h (FPIA1 and FPIA2, respectively). FPIA1 and FPIA2 polymers were modified by N-alkylation reaction with an excess of iodomethane (molar relationship 1:10) at 70 °C for 7 days (MeFPIA1 and MeFPIA2, respectively). The main difference between both polymers is the degree of quaternization. This synthesis of the polymers and their characterization are well detailed in Cottet et al. (2021) [8]. In Figure 1, the structure of the synthesized polymers, MeFPIAx, can be observed considering a degree of esterification and quaternization of 100%.

Then, aqueous dispersions at 15% *w*/*w* based on gelatin and starch (4:1 mass ratio) were prepared in distilled water. Afterwards, 10, 15, and 20% *w*/*w* of the synthesized polymers (MeFPIA1 and MeFPIA2) regarding the weight of gelatin and starch were added. Glycerol 30% *w*/*w* was incorporated based on the dry weight of gelatin and starch. A control sample without the addition of the synthesized polymers was also prepared.

Dispersions composed of gelatin, starch, glycerol, and the synthesized polymers were stirred in a thermostatic bath at 70 °C for 20 min. The pH of the solutions was then adjusted to a target interval of 8–9 by adding a 1 M NaOH solution, which was significantly more concentrated than the one used in the previous study, which was crucial for obtaining films with distinct properties. This pH interval was chosen because it improves the solubility of MeFPIAx and because prior studies have shown that gelatin can crosslink with various polyphenols, forming insoluble hydrogels when the dispersion pH is close to 9 [13]. This approach also opens up the possibility of incorporating a natural antioxidant agent, such as gallic acid, into the formulation in the future. In this case, the addition of the alkali appeared to prompt rapid crosslinking within the system. Therefore, the dispersion was immediately poured onto Petri dishes and dried for one week at room temperature. The dried films were peeled from the plates and conditioned for one week at 53% relative humidity (r.h.) provided by a saturated solution of Mg(NO_3_)_2_ at 25 °C, prior to their characterization. Table 1 shows the names and description of each prepared film; as mentioned, those without polymer were considered control films (GS).

### 2.3. Characterization of Active Films

#### 2.3.1. Fourier Transform Infrared Spectroscopy with Attenuated Total Reflectance (FTIR-ATR)

The analysis was performed on all films using an IR Affinity-1 Shimadzu spectrophotometer (Shimadzu Co., Kyoto, Japan) equipped with an attenuated total reflectance (ATR) module with a diamond crystal tip (GladiATR, Pike Technologies, Fitchburg, WI, USA). The scan covered a wavenumber range of 400 to 4000 cm^−1^, with 48 scans, 4 cm^−1^ resolution, and Happ-Genzel apodization. Spectra were obtained in duplicate.

#### 2.3.2. Differential Scanning Calorimetry (DSC)

Approximately 5 mg of each sample was placed into Tzero aluminum pans and sealed with hermetic lids. Studies were conducted using a Differential Scanning Calorimeter (TA Instruments Q200, New Castle, DE, USA). The first heating DSC scan was conducted from 30 °C to 180 °C at a rate of 10 °C min^−1^, with the main objective of eliminating the thermal history. Then, the samples were cooled down to −80 °C at a rate of 10 °C min^−1^. Finally, the second heating DSC scan was carried out from −80 °C to 180 °C at a rate of 10 °C min^−1^. Analyses were performed in duplicate and peak temperature and glass transitions were determined using TA Universal Analysis software (v4.5, TA Instruments, New Castle, DE, USA) at the mid-point.

#### 2.3.3. Thermogravimetric Analysis (TGA)

The mass loss in samples as a function of temperature was registered by themogravimetry in a Q-500 thermogravimetric analyzer (TA Instruments, New Castle, DE, USA). Approximately 7 mg of sample was weighed and analyzed by heating at 10 °C min^−1^ under inert nitrogen atmosphere (flow rates: 40 mL min^−1^ in balance and 60 mL min^−1^ in sample) from 30 °C to 700 °C. From TGA curves, initial degradation temperatures (T_i_) were calculated at 15% of mass loss; and the maximum degradation temperature of each degradation zone (T_max_) was obtained from the derivative of mass loss with respect to temperature. Determinations were performed in duplicates.

#### 2.3.4. Mechanical Properties

Mechanical properties were tested at room temperature using a Universal Test Instrument Megatest TC-500 series II (Megatest, Argentina) equipped with a 30 kgf cell load, and experiments were performed at 15 mm min^−1^. Before testing, rectangular samples (dimensions 50 mm × 10 mm) were cut for each formulation and conditioned at 53% r.h. provided by a saturated solution of Mg(NO_3_)_2_. The thickness of each rectangle was determined at five points using a digital micrometer (INSIZE Co., LTD, Suzhou New District, China, ±0.001 mm). The resulting stress–strain curves allowed for calculating the mechanical parameters: Young’s modulus (YM, MPa), tensile strength (TS, MPa), and elongation at break (EB, %).

#### 2.3.5. Water Vapor Permeability (P_w_)

The water vapor permeability (P_w_) of the films was determined by quantifying the water vapor flow through the films gravimetrically, following the ASTM E-E96 [19] standard with some modifications [20]. Firstly, the films were placed in acrylic cells containing a saturated solution of BaCl_2_, which provides an r.h. of 90% inside the cell. The cells were placed inside a desiccator containing a saturated solution of NaOH to provide an r.h. of 10%, at a constant temperature of 22 °C. A fan was placed over the films to maintain uniform air conditions inside the desiccator, following recommendations from previous authors [21]. The weights of the cells were recorded at the beginning of the experiment and at specified time intervals using a precision analytical balance (Precisa 125 A SCS, 10^−3^ g). Once the weight values over time were obtained, the P_w_ was calculated according to Equation (1).

(1) $$P_{w}=\frac{1}{A}\left(\frac{∆m}{∆t}\right)\frac{L}{{∆p}_{w}}$$

where A is the effective area of the exposed film and Δm/Δt is the slope of a linear regression of weight versus time. L is the film thickness and Δp_w_ = (p_w2_ − p_w1_) (in Pa units) is the differential water vapor partial pressure across the film; p_w1_ is the partial pressure of water vapor at the film surface outside the cup and p_w2_ is the partial pressure of water vapor at the film surface inside the cup. Experiments were performed in duplicate.

#### 2.3.6. Swelling Properties

The swelling of the polymeric films was evaluated in phosphate-buffered saline (PBS, pH 7.4) solution at 25 °C and 37 °C. Disks of approximately 1 cm were cut, weighed (around 40 mg), and immersed in containers with 20 mL of PBS. Swelling was monitored by measuring the weight gain due to the incorporation of the solution in the matrix during the immersion time. At specified time intervals, the disks were removed and excess solution was carefully removed with filter paper, weighed, and returned to the containers. This procedure was repeated until no further solution gain (constant weight) was observed or until disk integrity loss was observed. The assay was conducted in triplicate. The swelling process was expressed as solution absorption, s(t), at time t in units of g of solution per 100 g of dry film, and was calculated using Equation (2).

(2) $$s_{\left(t\right)}=\frac{W_{t}-W_{0}}{W_{0}}\times 100$$

where W_t_ is the weight of the swollen disk at time t and W_0_ is the weight of the conditioned disk at the beginning of the experiment.

The solution absorption s as a function of time t was fitted with a first-order kinetic model using a biexponential function that considers two stages of solution absorption [21], as shown in Equation (3).

(3) $$s_{\left(t\right)}=s_{0}+{\Delta s}_{1}\left(1-\exp\left(-\frac{t}{\tau_{1}}\right)\right)+{\Delta s}_{2}\left(1-\exp\left(-\frac{t}{\tau_{2}}\right)\right)$$

where s(t) is the solution absorption; s_0_ is the initial solution content (s_0_ = 0 under experimental conditions); Δs_1_ and Δs_2_ are the solution absorption related to sorption processes 1 and 2, respectively; and τ_1_ and τ_2_ are the time constants for processes 1 and 2, respectively. The equilibrium solution absorption s_ꝏ_ was calculated using Equation (4).

(4) $$s_{ꝏ}={\Delta s}_{1}+{\Delta s}_{2}$$

#### 2.3.7. Evaluation of Antioxidant Properties by ABST^•+^ and DPPH Assays

The antioxidant activity of the films was evaluated by applying the ABTS method. For the evaluation of the antioxidant activity by the ABTS method, the radical ABTS^•+^ was formed in aqueous solution by mixing 7 mM of ABTS and 2.45 mM K_2_S_2_O_8_ (solution A). This mixture was left for 16 h in the dark, and after this period of time, solution A was diluted in milliQ water (solution B) until the absorbance of the solutions was adjusted to 0.70 ± 0.02 at a wavelength of 734 nm. Disks of about 10 mg of each sample were cut and placed in Eppendorf tubes with 1 mL of solution B. After 5 min of reaction in the dark with slight agitation, the absorbance of the solution was measured at a wavelength of 734 nm. Each condition was tested at least in duplicate.

Additionally, the antioxidant activity was analyzed using the stable radical DPPH, as each assay can detect different action mechanisms. Therefore, by employing both methods, a more comprehensive view of the antioxidant activity of the films was obtained. This method relies on color decay when the odd electron of the nitrogen atom in the DPPH radical is reduced by receiving one hydrogen atom from the antioxidant compounds. DPPH was prepared in absolute ethanol at a concentration of 1 mM and allowed to react for one hour in darkness with slight agitation; the absorbance of the solution was measured at a wavelength of 517 nm. Each measurement was performed at least in duplicate.

The antioxidant activity of the different films, by using both methods, was calculated by the radical scavenging activity percentage (RSA(%)), as described in Equation (5).

(5) $$RSA(\%)=\frac{blank\;absorbance-sample\;absorbance}{blank\;absorbance}\times 100$$

where blank corresponds to ABTS solution B absorbance without film, and ethanol in the case of the DPPH method.

#### 2.3.8. Antimicrobial Activity of Films

The methodology developed was based on the ASTM E2149-01 method [22]. The antimicrobial activity of the films was evaluated against Gram-positive *Staphylococcus aureus* bacteria strains (*S. aureus*, ATCC 25923). The microorganism isolate was incubated in sterile nutrient agar plates for 24 h at 37 °C. Then, a bacteria suspension was prepared in tubes with sterile PBS (pH 7.4) and diluted until a final concentration of 10^5^ CFU/mL was obtained. Before testing, the films were conditioned at 53% r.h. Then, samples were cut into squares of 1 cm^2^ and placed in Eppendorf tubes with 1 mL of the inoculum solution. They were incubated at 37 °C while shaking at 100 rpm. Aliquots were taken and total cell counts were made at 0 and 24 h of contact time. A control of the inoculum solution was carried out (without films) and those films that did not contain MeFPIAx (their inactivity was confirmed) were also considered as control films. The percentage of bacterial reduction (%) was calculated using Equation (6).

(6) $$Bacterial\;reduction\left(\%\right)=\frac{B-A}{B}\times 100$$

where A is CFU/mL for the film after 24 h contact time, and B is CFU/mL at the initial time.

### 2.4. Statistical Analysis

Data were expressed as means ± standard deviation. The values corresponding to mechanical properties, water vapor permeability, and swelling capacity were analyzed by analysis of variance (ANOVA) tests using PSPP Software, version 0.9.0-g745ee3. Tukey’s honest significance test was applied to find means that were significantly different from each other, with a significance level of *p* < 0.05.

## 3. Results and Discussion

### 3.1. Fourier Transform Infrared Spectroscopy with Attenuated Total Reflectance (FTIR-ATR)

**Figure 2:**
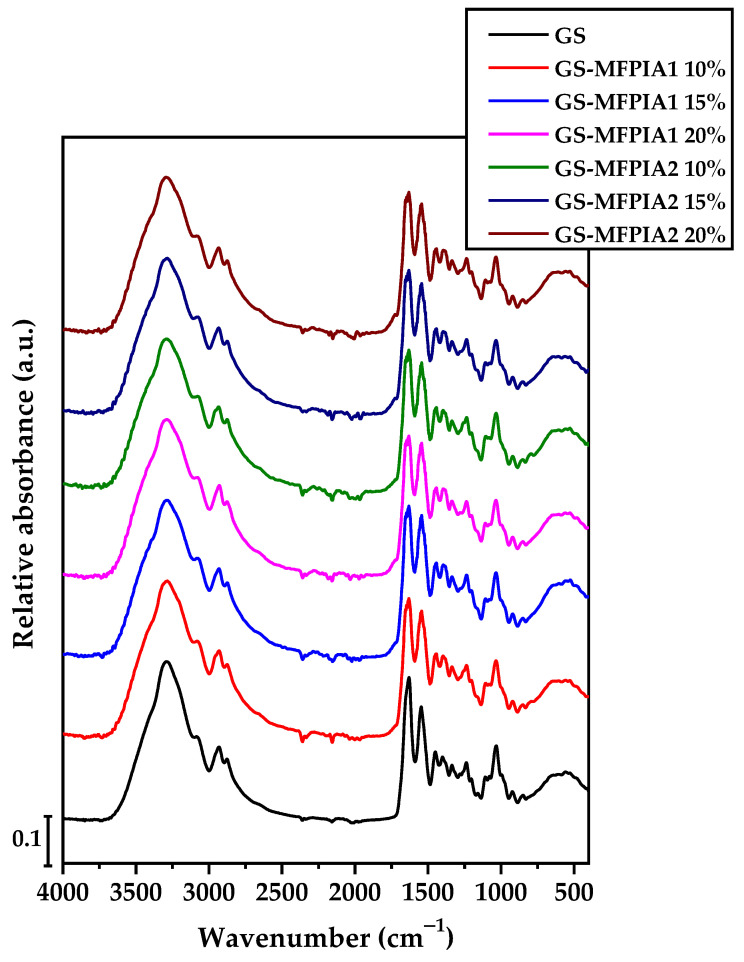
Fourier Transform Infrared Spectrum with Attenuated Total Reflectance (FTIR-ATR) of gelatin- and starch-based films.

The infrared spectra display the absorption bands of the gelatin- and starch-based films in the presence and absence of different concentrations of MeFPIAx, as can be seen in Figure 2.

Firstly, in the range from 3750 to 3000 cm^−1^, a broad band appears, which is associated with the absorption of hydroxyl groups (-OH) from remaining water, glycerol and starch, as well as the N-H bonds of the amino group of gelatin [23,24]. The bands found in the range of 3000 to 2800 cm^−1^ correspond to the bending of the C-H bond of methyl and methylene functional groups. A small shoulder/peak ascribed to the absorption of the C=O group is identified in the region around 1700–1725 cm^−1^ in samples containing the quaternized polymers. However, the characteristic signals of the MeFPIAx polymers overlap with the signals of gelatin and starch; therefore, no significant differences were observed among the different spectra. The spectrum presented two bands, one at one on 1634 cm^−1^ is ascribable to carbon-to-oxygen (C=O) stretching within the carboxylic group (amide I), and the peak at 1551 cm^−1^ is correlated to the N–H bond (amide II) stretching of gelatin [25,26]. Finally, in the range between 1200 and 800 cm^−1^, bands assigned to vibrations of C-O-C, C-C, C-OH, and C-H bonds were found. These bands could be associated with the vibrational movements of the functional groups corresponding to polysaccharides [26,27].

### 3.2. Differential Scanning Calorimetry (DSC)

**Figure 3:**
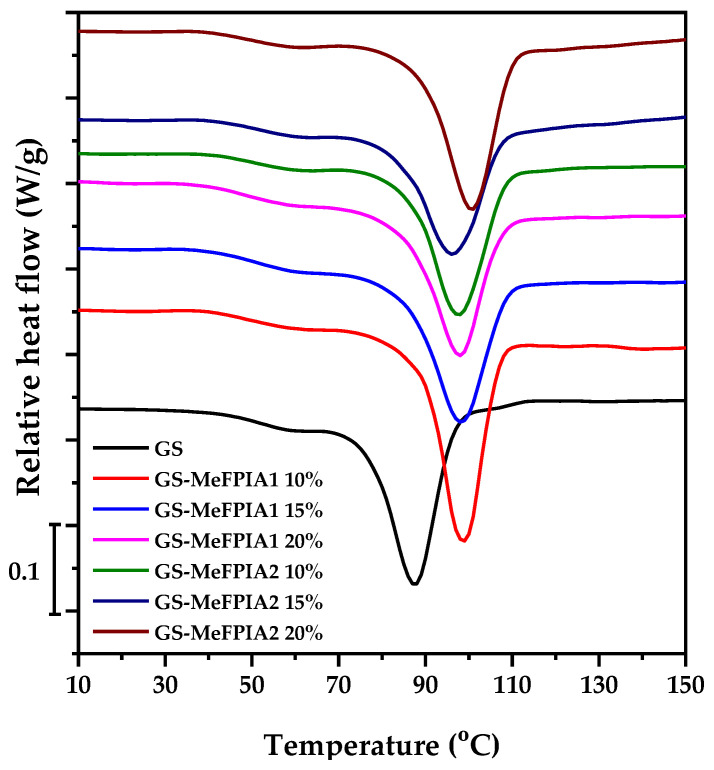
DSC thermograms of gelatin- and starch-based films.

Prior to the test, the film samples were dehydrated in a desiccator containing silica gel to identify thermal events independent of water content, as water acts as a plasticizing agent in the films [28,29]. Figure 3 shows the corresponding total heat flow thermograms of the samples. The identified glass transition temperatures, T_gs_, are of utmost importance for selecting the processing and storage conditions of the films and defining their potential applications. Two heating cycles were performed; the first heating scan cycle was carried out in order to eliminate thermal history in the sample because starch and gelatin properties in the solid state depend critically on the thermal history [30]. Their corresponding transition temperatures are presented in Table 2. The DSC curves of these films showed, in all cases, only one T_g_ followed by an endothermic peak (T_peak_), in concordance with previous studies showing similar results [30,31]. T_g_ is the temperature at which the material changes from the glassy state to the elastomeric state for a given heating rate, and values ranging between 64 and 68 °C were obtained. The highest values compared to the control film were found in the films containing polymer, both MeFPIA1 and MeFPIA2; however, no significant differences were observed with the incorporation of different percentages. This behavior is possibly due to the increase in electrostatic forces and hydrogen bonding interactions that occur between the gelatin and starch macromolecules with the carboxyl groups of the polymers. The increase in intermolecular forces between the polymer chains leads to a decrease in free volume and system mobility, hindering the transition to the elastomeric state [32]. Therefore, as the movement becomes more restricted, the T_g_ shifts towards higher values.

Regarding the presence of an endothermic peak, it is characteristic of the relaxation process (physical aging) and occurs due to the fact that during the first heating stage in a DSC experiment, the material recovers the energy released during storage where gelatin molecules could undergo molecular changes, such as aggregation [31]. This phenomenon is due to the decrease in specific volume when the material relaxes, and to the decrease in molecular mobility resulting from that relaxation, causing an energy recovery at a higher temperature [33]. When MeFPIAx was added, there was an increase in intermolecular forces between polymer chains (lower specific volume and lower molecular mobility), though the temperature of the endothermic peak shifts towards higher values. This process was confirmed with the second heating run where such a transition disappeared because the material’s thermal history was eliminated. On the other hand, previous studies have attributed this endothermic peak to the presence of crosslinks in gelatin, which consist of regions where the protein has reverted to the triple-helix structures similar to collagen. The random protein coils undergo partial renaturation back into triple helices during the gelling and subsequent drying of gelatin. The degree of renaturation, which is linked to the triple-helix content, is an important physical characteristic of the gelatin-based films dominating their thermal performance. The triple helices could act as physical crosslinks; increasing the number of crosslinking points in polymer networks resulted in more hindered chain movements that may produce an increase in the peak temperature [30,34].

### 3.3. Thermogravimetric Analysis (TGA)

**Figure 4:**
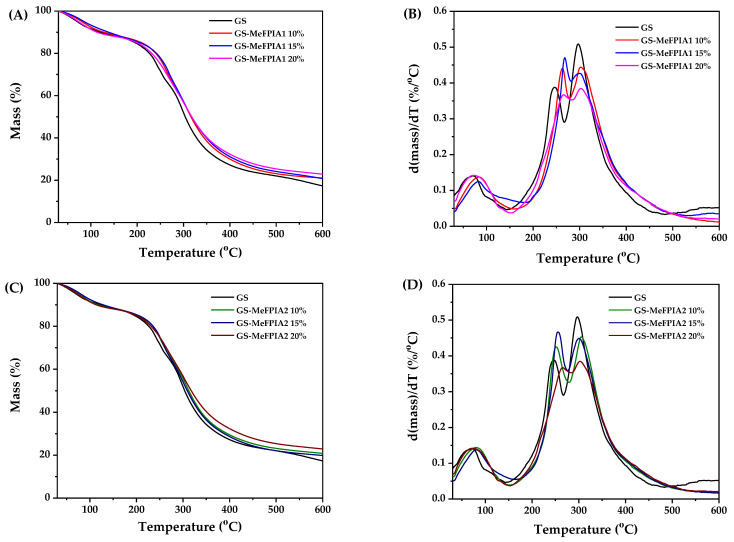
Thermogravimetric curves: (**A**,**C**) mass loss as function of temperature; (**B**,**D**) derivative of percentage mass as function of temperature.

The results of the thermogravimetric analyses of the developed films are presented in Figure 4. The initial degradation temperature after water loss (T_i_) was determined as the temperature at which 15% of mass loss occurs, while the temperatures of thermal degradation (T_max_) for each stage were determined from the peaks of the derivative curves (d(mass%)/dT). 

The data for the initial and thermal degradation temperatures (T_i_ and T_max_) are presented in Table 3. Three main degradation zones could be observed within a temperature range from 30 to 700 °C with a residue of 20%. The first thermal degradation zone occurred around 100 °C in all films, which could be attributed to the loss of free and bound water adsorbed in the material [35]. The second zone was observed in the range of 150–250 °C. This event possibly involves the onset of partial degradation of proteins associated with protein breakage and peptide chain rupture, as well as loss of glycerol plasticizer [36]. Additionally, the synthesized polymers begin to degrade, which explains why thermal degradation in this temperature range is more significant for samples with MeFPIAx compared to the control film (GS).

Finally, a third zone (around 300 °C) was associated with polysaccharide degradation, which corresponded to a complex process including depolymerization and dehydration of the saccharide rings, and the complete degradation of proteins and synthesized polymers [30,37,38]. It is noteworthy that 50% of the initial mass was retained at temperatures up to 300 °C. The residual mass content of the sample is approximately 20% in all cases [39].

Regarding the Ti values, it is revealed that the incorporation of MeFPIAx led to an increase in these parameters, which may be due to the fact that MeFPIAx could promote the formation of a stable network through inter- and intra-hydrogen bonding among all components. Due to stronger interactions, more thermal energy is needed for the dissociation of polymer molecules from the ordered structure of composite films [27]. This indicates that the films are more thermally resistant, confirming an increase in the interactions between their components. These results suggest that the material could be processed at temperatures close to 190 °C without degradation.

### 3.4. Mechanical Properties

Although the use of natural polymers is beneficial from an eco-friendly point of view, there are some inherent limitations resulting from their properties, such as tensile strength, that need to be improved [40]. Polymer blending is an interesting method for developing new materials that combines different components with certain properties, obtaining a synergistic effect in the final material [41]. While gelatin-based films provide lower mechanical properties compared to synthetic ones, gelatin films become fragile under specific drying conditions, thereby becoming rigid and brittle and exhibiting poor elongation at break. Also, gelatin films may dissolve, swell, or disintegrate upon contact with moisture. Thus, modification of gelatin films with suitable polymers is essential to minimize those limitations [42]. The combination of gelatin with other natural polymers with different characteristics, such as whey proteins, starch, chitosan, or pectin, is a good strategy for the development of films with improved mechanical properties [43].

The values corresponding to tensile strength (TS), Young’s modulus (YM), and elongation at break (EB) of the different films are presented in Table 4. Films made with different blend ratios based on gelatin have been reported to have both higher and lower mechanical parameter values than those obtained in the present research. Also, data comparison with other studies is difficult due to experimental variables like gelatin source (whether it is bovine, porcine, chicken, or fish) and the formulation and film conditioning methods [42,44,45]. An increase in TS and MY values was observed, and consequently, a decrease in EB% values, upon adding different concentrations of MeFPIA1 and MeFPIA2. These differences showed statistically significant variation (*p* < 0.05). Additionally, the TS and EB of the films are inversely related. The presence of MeFPIAx in the filmogenic solution could increase interactions with gelatin and starch, forming hydrogen bonds between the hydroxyl groups of starch, the carboxylic groups of the polymer, and the amino groups of gelatin [26]. In this way, intermolecular forces along the polymeric chains were enhanced, resulting in higher TS and reduced flexibility and mobility of the chains. Table 4 shows that the incorporation of MeFPIAx led to a rise in YM as the concentration of both polymers increased, indicating improved stiffness of the material. This enhancement was significantly observed with the addition of 20% MeFPIA1. Additionally, elongation exhibited an inverse correlation with the rising concentrations of polymer. The difference between films with both functionalized polymers is that MeFPIA1 has a higher number of free carboxylic acid groups capable of interacting with protein chains. Comparing the control sample GS with the control previously reported [8], as well as formulations GS-MeFPIA1 10% and GS-MeFPIA2 10%, it is possible to observe differences in the results related to differences in the formulation and procedure for the incorporation of the alkali. This work presented samples with higher YM and TS values, which may be related to the crosslinking observed during the preparation of the dispersion.

### 3.5. Water Vapor Permeability (P_w_)

Given that gelatin contains a wide range of hydrophilic amino acids, films with high protein content could absorb more water molecules from the environment [20]. Additionally, considering that a proportion of modified starch is present in the formulation, which absorbs a large amount of water, there is a strong affinity for water molecules in the formulated films. As a result, the diffusion of water through these films is high, resulting in materials with high water vapor permeability [45]. The P_w_ data are presented in Table 5, and as can be seen, the incorporation of higher concentrations of MeFPIAx polymer reduced these values. This effect is more substantial with the incorporation of MeFPIA1. While the polymers increase the content of hydrophilic groups in the matrix, they also increase interactions between the different components of the films, which is reflected in a higher water vapor barrier. By reinforcing the matrix, the polymeric chains have less mobility, making it more difficult for water vapor to pass through them. Comparatively with previous analysis [8], these new formulations are able to reduce their P_w_ values by 50%. This deviation can be explained only by differences in the procedure used to prepare the films. A 0.1 M NaOH solution dilutes and softens the structure, increasing chain mobility (as reflected in the mechanical properties) and water vapor permeability. In this study, the use of a more concentrated NaOH solution (1 M) reduced the flexibility of the polymeric structure, which in turn decreased water mobility within the structure. As a result, P_w_ decreased with the more concentrated NaOH solution, while the presence of MeFPIAx further strengthened interactions, amplifying this effect.

### 3.6. Evaluation of Swelling Capacity of Films and Their Kinetics

**Figure 5:**
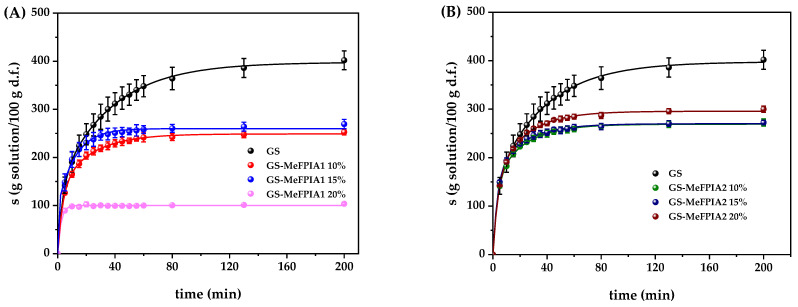
Swelling curves of the different films at 25 °C in PBS: (**A**) GS, GS-MeFPIA1 10%, GS-MeFPIA1 15%, GS-MeFPIA1 20%; (**B**) GS, GS-MeFPIA2 10%, GS-MeFPIA2 15%, GS-MeFPIA2 20%.

Determining the swelling of films is an important factor as it measures the efficiency of a film to maintain its structural integrity in an aqueous environment. Figure 5 shows the swelling behavior of the different films in PBS at 25 °C. While the interest in blending polysaccharides and proteins relies on the complementary advantages of these components [46], gelatin and starch are highly hydrophilic polymers [47]. Therefore, films with a high water absorption capacity are formed due to the large number of water-affine groups present in the matrix [42]. Thus, control films without MeFPIAx showed a higher degree of swelling and were the weakest, as they lost their integrity over the tested time. However, films with MeFPIAx showed a lower degree of swelling compared to the control and were the most stable. With the incorporation of the polymer, as its concentration in the blend increased, the number of intermolecular interactions increased, causing the material to lose elasticity by reducing the spaces between the polymeric chains. This makes the material less flexible and, therefore, decreases its ability to deform and swell [48,49].

In Figure 5A, it can be clearly observed that incorporating 10% and 15% of MeFPIA1 results in reductions in swelling values, with no significant differences between both percentages. However, upon incorporating 20%, swelling values are considerably reduced, possibly due to a greater number of carboxylic groups that can interact with gelatin and starch. This result was expected, since the films exhibited the highest YM values and the lowest EB values.

In Figure 5B, it can be observed that incorporating 10% and 15% of MeFPIA2 also results in a reduction in swelling values compared to control films, with very similar values recorded. However, upon incorporating 20%, these values were slightly higher, although no significant differences were observed. It is important to note that films at all three concentrations presented similar MY values, which are related to the flexibility of the material.

**Figure 6:**
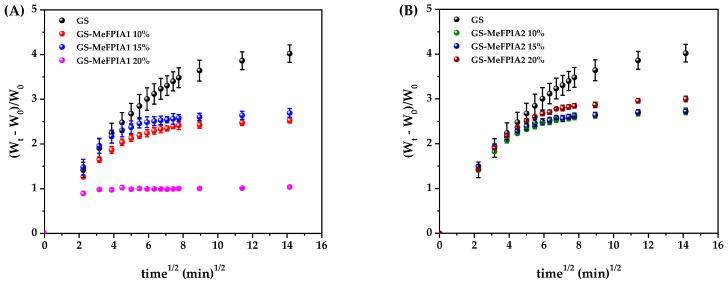
Representation of (W_t_ − W_0_)/W_0_ versus t^1/2^ at 25 °C in PBS: (**A**) GS, GS-MeFPIA1 10%, GS-MeFPIA1 15%, and GS-MeFPIA1 20%; (**B**) GS, GS-MeFPIA2 10%, GS-MeFPIA2 15%, and GS-MeFPIA2 20%.

When plotting (W_t_ − W_0_)/W_0_ against t^1/2^ in the initial swelling stages, the Fickian behavior can be analyzed. The corresponding graphs are shown in Figure 6. As can be observed, the water uptake curves of the film solution for all formulations were nonlinear up to a 60% increase in hydrogel mass. Therefore, as seen in the figure, most formulations did not follow one-dimensional Fickian diffusion, so the values of *n* are different from 0.5 and the data must be adjusted for a non-Fickian or anomalous diffusion process. Consequently, the experimental data were adjusted using a biexponential function. This model fit the data quite well according to the behavior of the films during swelling (see the lines in Figure 5A,B) and the kinetic swelling parameters of the model are showed in Table 6.

### 3.7. Evaluation of Antioxidant Properties by ABST^•+^ and DPPH Assays

**Figure 7:**
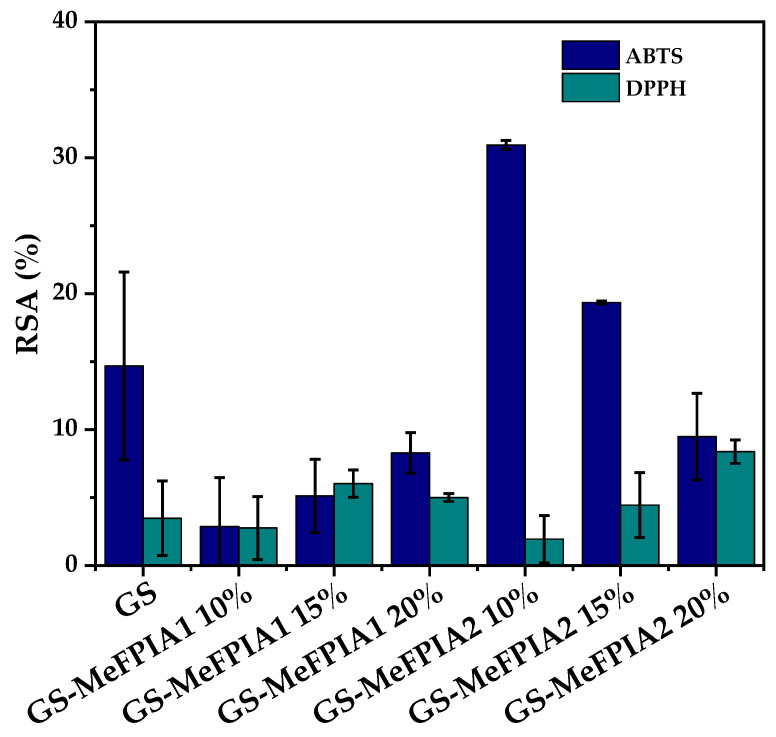
Antioxidant activity of films by ABTS and DPPH methods.

The antioxidant capacity of the materials was evaluated through two of the most commonly used methods to gain a better understanding of the film properties. Each method is based on different chemical principles and provides complementary information about the antioxidant capacity of the materials. Figure 7 shows the results of the antioxidant activity of the films using the ABTS and DPPH methods. According to the results obtained, it is worth noting that GS films without MeFPIAx exhibited antioxidant activity that could be assigned to the antioxidant capacity of amino acids such as proline and glycine due to the presence of gelatin [50]. These results are consistent with previous studies that also reported similar findings, mainly attributing the antioxidant activity of gelatin-based films to the peptide fraction [51]. Upon incorporating MeFPIA1, it was observed that the RSA (%) value decreased compared to the control in the ABTS assay, which could be due to an interaction between the polymers where those groups reacting with ABTS are compromised. On the other hand, the incorporation of 10% MeFPIA2 showed an increase in the RSA (%) value. As for the DPPH assay, a slight increase in the RSA (%) value was observed with the addition of MeFPIAx, although this increase was not very substantial. The presence of quaternized polymers in the formulation enhances the antioxidant activity of films.

### 3.8. Antimicrobial Activity of Films

**Figure 8:**
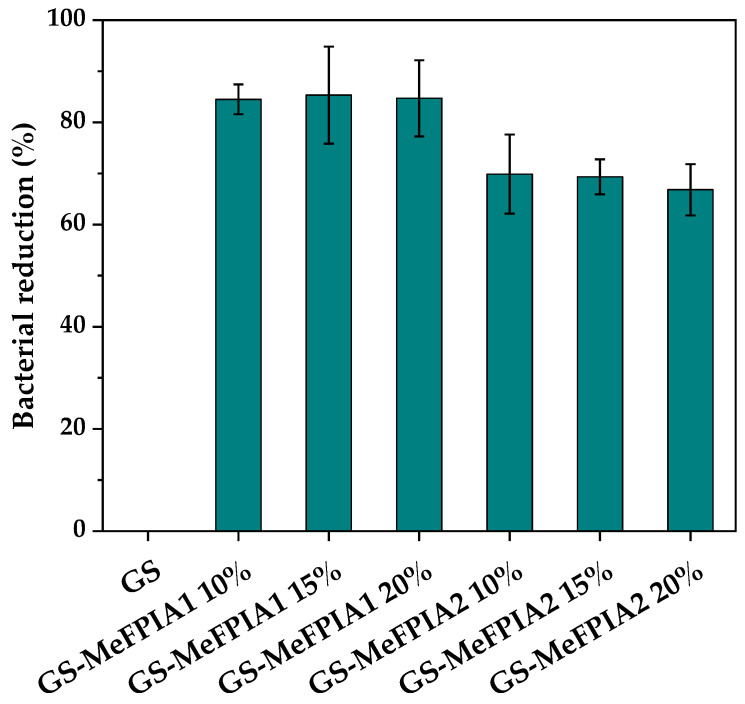
Percentage of bacterial reduction against *S. aureus* of different films.

The results presented in Figure 8 show the bacterial reductions (%) in the developed films after a 24 h incubation period at 37 °C with slight agitation in contact with *S. aureus* bacteria. According to the reduction percentages obtained, it is observed that all films except the control sample (without MeFPIAx) exhibited antimicrobial activity, as expected, due to the inability of gelatin and starch to inhibit bacterial growth [52,53]. Despite the continuous development of more potent antibiotics, antimicrobial polymeric materials, especially cationic polymers, present significant advantages. As mentioned previously, one method to fabricate surfaces that are biocidal active is the incorporation of an antimicrobial polymer into a matrix because of its intrinsic nature and/or due to biocidal groups and segments incorporated into the chain backbone and/or as side chains. These materials have the ability to kill some antibiotic-resistant pathogens due to their mechanism of action. The antimicrobial activity derives from their cationic character, which can bind to the negatively charged membranes of the microbes that are attracted to it, leading to their electrostatic instability and cell lysis [8,54]. The antimicrobial capacity of the materials was significantly improved with the incorporation of MeFPIA1 and MeFPIA2 by approximately 80% and 70%, respectively, through the increase in positive charge density. Previous studies have shown the development of new polymeric systems prepared by free radical polymerization, which can be tuned into effective and broad-spectrum antimicrobials by post-modification (quaternization) due to the versatility of thiazole pendant groups [2,5,6]. Another specific case studied the degree of cell elimination against *Candida parapsilosis* microorganisms upon exposure to antimicrobial films based on a blend of polyacrylonitrile (PAN) and antimicrobial methacrylic copolymers with attached cationic moieties (1,3-thiazolium and 1,2,3-thiazolium side-chain groups), P(AN-*co*-MTA). The biocidal cell-killing efficiency was significantly improved, reaching almost 100% [53]. Therefore, there is no doubt about the benefits and effectiveness of quaternary polymers against microorganisms [55]. On the other hand, gelatin and starch are considered as promising matrices because both possess excellent film-forming properties and they have been used in several systems such as injectable hydrogels, drug delivery systems, and scaffolds, among other applications [56,57]. Studies revealed that starch-based films incorporated with citric acid showed that 98–99% of food borne bacteria growth and 87–99% of fungal growth can be inhibited [58]. It was found that antibacterial starch-based materials were most widely used in packaging, followed by medicine [59]. Also, adding essential oils into gelatin-based film can enhance the antibacterial activity of the film against Gram-positive and Gran-negative bacteria, and that the antimicrobial activity of films was found to be proportional to essential oil content [24]. Although the results obtained in the present study were lower than some reported values, it should be considered that one of the major advantages of incorporating quaternary polymers, as mentioned earlier, is that, unlike release materials, the antimicrobial agents anchored to the film matrix prolong the antimicrobial activity because they are not easily released into the surrounding medium, thereby extending the antimicrobial activity of the material and preventing microbial resistance due to their mode of action.

It should be noted that no significant differences were observed among the different concentrations of MeFPIAx evaluated. It is possible that, although the concentration of quaternary polymer increases, these functional groups may not be available due to increased interactions among the different components. Therefore, the election of 10% MeFPIAx would be the most suitable for incorporation into these matrices, as antimicrobial properties are not further improved with a higher polymer content.

## 4. Conclusions

The incorporation of antimicrobial quaternary polymers into gelatin- and starch-based films demonstrated promising results for developing materials with enhanced properties. The films exhibited improved mechanical properties, with higher tensile strength (TS) and a higher Young’s modulus (YM), with values close to 20 MPa and exceeding 250 MPa, respectively. Furthermore, the addition of MeFPIAx increased the glass transition temperature from 29 to 65 °C and the initial degradation temperatures, indicating the greater thermal resistance of the films. Due to increased interaction, water molecule absorption during swelling was clearly reduced from approximately 400% to 100%, and a reduction in water vapor permeability was also observed. These findings suggest that variations in the percentages of incorporated quaternary polymers did yield significant differences in these evaluated properties. The films exhibited slight antioxidant activity that was higher than without quaternized polymers. In addition, significant antimicrobial activity was observed, achieving bacterial reduction values greater than 70% with the incorporation of both polymers against *Staphylococcus aureus*; this was the case for MeFPIA1, which had a lower degree of modification while presenting higher activity. No major differences were observed at different concentrations but the best results were achieved when MeFPIA1 is incorporated. Finally, in conclusion, it can be said that a balance needs to be found between selecting the optimal concentration of MeFPIAx and the desired final properties.

Another possible application for these films could be as wound healing dressings; therefore, studies on films’ cytotoxicity should be performed using mammalian cell growth. On the other hand, a general limitation of these materials is that, although MeFPIAx can enhance the performance of gelatin- and starch-based films, both polymers are highly hydrophilic, which is a drawback for packaging applications. Therefore, with this in mind, another implementation would be the chemical modification of synthesized FPIA through alkylation reactions with longer alkyl chains (butyl, hexyl, or octyl) to impart greater hydrophobicity to the material.

## Figures and Tables

**Table 1:** Description of gelatin- and starch-based films.

Sample Names	Description
GS	Films based on gelatin, starch, and glycerol
GS-MeFPIA1 10%	Films based on gelatin, starch, glycerol, and MeFPIA1 10%
GS-MeFPIA1 15%	Films based on gelatin, starch, glycerol, and MeFPIA1 15%
GS-MeFPIA1 20%	Films based on gelatin, starch, glycerol, and MeFPIA1 20%
GS-MeFPIA2 10%	Films based on gelatin, starch, glycerol, and MeFPIA2 10%
GS-MeFPIA2 15%	Films based on gelatin, starch, glycerol, and MeFPIA2 15%
GS-MeFPIA2 20%	Films based on gelatin, starch, glycerol, and MeFPIA2 20%

**Table 2:** Transition thermal temperatures of gelatin- and starch-based films. Temperature error: ±1 °C.

	First Heating		Second Heating
Sample Names	T_g_ (°C)	T_peak_ (°C)	T_g_ (°C)
GS	65	88	29
GS-MeFPIA1 10%	67	99	56
GS-MeFPIA1 15%	66	98	65
GS-MeFPIA1 20%	66	98	60
GS-MeFPIA2 10%	68	98	39
GS-MeFPIA2 15%	65	96	45
GS-MeFPIA2 20%	64	101	49

**Table 3:** Initial and maximum degradation temperatures- of gelatin and starch-based films. Temperature error: ±1 °C.

Sample Names	T_i_ (°C)	T_max 1_ (°C)	T_max 2_ (°C)
GS	196	241	297
GS-MeFPIA1 10%	211	265	300
GS-MeFPIA1 15%	207	256	296
GS-MeFPIA1 20%	201	265	292
GS-MeFPIA2 10%	204	245	299
GS-MeFPIA2 15%	204	255	300
GS-MeFPIA2 20%	201	264	296

**Table 4:** Mechanical parameters of gelatin- and starch-based films.

Sample Names	TS (MPa)	YM (MPa)	EB (%)
GS	10.8 ± 0.6 ^a^	114.1 ± 7.7 ^a^	86.5 ± 5.2 ^a^
GS-MeFPIA1 10%	20.1 ± 2.0 ^b^	266.3 ± 13.1 ^b^	68.2 ± 7.7 ^b,d^
GS-MeFPIA1 15%	19.2 ± 1.1 ^b^	261.8 ± 37.1 ^b^	61.8 ± 5.0 ^b,d^
GS-MeFPIA1 20%	18.8 ± 1.0 ^b^	342.7 ± 52.2 ^c^	36.3 ± 7.8 ^c^
GS-MeFPIA2 10%	21.4 ± 1.4 ^b,c^	259.0 ± 12.7 ^b^	70.3 ± 3.3 ^b^
GS-MeFPIA2 15%	23.2 ± 1.1 ^c^	271.2 ± 17.3 ^b^	71.2 ± 4.3 ^b^
GS-MeFPIA2 20%	20.8 ± 1.1 ^b,c^	284.7 ± 21.2 ^b^	59.8 ± 4.3 ^d^

The same letters in the data reported in a column mean non-significant differences (*p* ≥ 0.05).

**Table 5:** Water vapor permeability values of gelatin- and starch-based films.

Sample	Permeability (10^−10^ g s^−1^ m^−1^ Pa^−1^)
GS	4.89 ± 0.05 ^a,e^
GS-MeFPIA1 10%	4.60 ± 0.03 ^b^
GS-MeFPIA1 15%	4.53 ± 0.03 ^c^
GS-MeFPIA1 20%	4.30 ± 0.03 ^d^
GS-MeFPIA2 10%	4.81 ± 0.09 ^a,e^
GS-MeFPIA2 15%	4.98 ± 0.08 ^a^
GS-MeFPIA2 20%	4.76 ± 0.09 ^e^

The same letters in the data reported in a column mean non-significant differences (*p* ≥ 0.05).

**Table 6:** Kinetic swelling parameters for the different gelatin- and starch-based films.

Sample Names	s_ꝏ_ (g/g)	Δs_1_ (g/g)	τ_1_ (min)	φ_1_	Δs_2_ (g/g)	τ_2_ (min)	φ_2_
GS	398 ± 12 ^a^	140 ± 7 ^a^	3.4 ± 0.3 ^a^	0.35	258 ± 5 ^a^	37 ± 1 ^a^	0.65
GS-MeFPIA1 10%	249 ± 17 ^b^	133 ± 9 ^a^	3.4 ± 0.4 ^a^	0.53	116 ± 8 ^b^	22 ± 2 ^b^	0.47
GS-MeFPIA1 15%	265 ± 17 ^bc^	186 ± 9 ^b^	4.1 ± 0.3 ^a^	0.70	79 ± 8 ^c^	24 ± 3 ^b^	0.30
GS-MeFPIA1 20%	100 ± 1 ^d^	100 ± 1 ^c^	2.2 ± 0.2 ^b^	1	--	--	--
GS-MeFPIA2 10%	269 ± 16 ^bc^	150 ± 8 ^ad^	3.3 ± 0.3 ^a^	0.56	119 ± 8 ^b^	22 ± 2 ^b^	0.44
GS-MeFPIA2 15%	270 ± 16 ^bc^	170 ± 9 ^bd^	3.5 ± 0.3 ^a^	0.63	100 ± 8 ^bc^	22 ± 2 ^b^	0.37
GS-MeFPIA2 20%	295 ± 22 ^c^	152 ± 12 ^da^	3.8 ± 0.4 ^a^	0.52	143 ± 11 ^d^	22 ± 2 ^b^	0.48

The same letters in the data reported in a column mean non-significant differences (*p* ≥ 0.05).

## Data Availability

The original contributions presented in the study are included in the article, further inquiries can be directed to the corresponding author.
